# A Study of the Correlation Between Screen Time and Hypertension Among Young Adults in North India: A Cross-Sectional Analysis

**DOI:** 10.7759/cureus.51667

**Published:** 2024-01-04

**Authors:** Maslahuddin Hayat Ahmad Alhaque Roomi, Abhishyant Srivastava, Narinder Girdhar, Chaitannya Jha, Shashwat Thakur

**Affiliations:** 1 Internal Medicine, Dr. Baba Saheb Ambedkar Medical College & Hospital, New Delhi, IND; 2 Medicine, Dr. Baba Saheb Ambedkar Medical College & Hospital, New Delhi, IND

**Keywords:** screen time, blood pressure, body mass index (bmi), young adults, hypertension, cross-sectional analysis

## Abstract

Background: Hypertension is a major risk factor for coronary artery disease. Due to the increased accessibility of smartphones over the past decade, there has been an increase in the screen time of adolescents and young adults. However, the relationship between screen time and hypertension has not been adequately studied. Our study aims to find a correlation between screen time and blood pressure (BP) among young adults.

Methods: A cross-sectional analysis was performed on a sample of medical students (n = 210) from New Delhi, India. Participants' screen time was monitored over three weeks and BP was recorded using a standardized sphygmomanometer by auscultatory method. Exclusion criteria included known cases of hypertension (with or without ongoing treatment), smokers > five pack year, heavy alcoholics, and participants having sleep time of less than seven hours or more than nine hours per day. Screen time was correlated with BP readings using standard statistical methods.

Results: Participants with screen time >390 minutes (six hours and 30 minutes), >420 minutes (seven hours), and >480 minutes (eight hours) had higher odds of elevated BP (OR: 1.86, 95% CI: 1.05-3.30; OR: 1.86, 95% CI: 1.04-3.30; OR: 1.87, 95% CI: 1.02-3.43, respectively) compared to students with screen time <390 minutes. The findings were consistent after excluding participants with high BMI based on the WHO and Asia-Pacific criteria, which also showed higher odds of elevated BP with screen time >390 minutes (OR: 3.21, 95% CI: 1.58-6.49 and OR: 3.92, 95% CI: 1.49-10.31, respectively). Regression analysis showed no significant linear correlation between screen time and BP (p > 0.05). However, a significant association was observed between BMI and elevated BP (p < 0.001).

Conclusion: This study revealed an association exists between screen time and BP.

## Introduction

Hypertension is a major risk factor for coronary artery disease. Worldwide, hypertension is estimated to cause 7.5 million deaths (WHO), i.e., about 12.8% of the total of all deaths. This accounts for 57 million disability-adjusted life years (DALYS) or 3.7% of total DALYS [1]. Although guidelines exist for screen time exposure in the paediatric population, there is still no guideline as to what an ideal screen time for an adult should be [2].

The Centers for Disease Control and Prevention showed that in 2021, 48.1% (119.9 million) of the population in the USA had hypertension [3], and young adults (National Health and Nutrition Examination Survey 2017-2022) make up 26.4% of all hypertensive population, which is a staggering number [4].

The overall prevalence of hypertension in India was found to be 29.8% (95% CI: 26.7-33.0) [5]. Geevar et al. in their study found that awareness and treatment of hypertension were significantly poor among younger adults compared to older adults, which shed light on the importance of diagnosing and treating hypertension in the young population [6].

The advent of the technological revolution has led to an increase in the use of electronic devices. Although these devices have the potential to enhance work efficiency, their excessive use has been shown to promote a sedentary lifestyle and poor sleeping habits. This lifestyle shift may have a detrimental impact on the psychological and physical well-being of an individual.

Prehypertension and hypertension are predicted to rise in the young population and strategies must be focused on specific areas to decrease the risk. Although many risk factors like obesity, smoking, alcohol, and amount of physical activity have independently been explored, there is not enough literature on screen time and its relationship with blood pressure (BP).

Despite various questionable health risks of excess screen time, there are few studies that identify these risk factors. Our study aims to find if any relationship exists between screen time and BP.

## Materials and methods

Sample size

A total of 450 students were enrolled in the Medical School from different batches. Stratified sampling was carried out based on the year of admission. The sample size was calculated by taking the anticipated frequency as 50%, the confidence limit as 95% significance, and precision as 5%. Formula: n = [Np (1-p)]/ [(d2/Z21-α/2*(N-1) +p*(1-p)]. Where, n = sample size, N = population size, p = prevalence, and d =precision. The required sample size was 208.

Study design

A cross-sectional analysis was carried out at Dr. Baba Saheb Ambedkar Medical College, New Delhi (n = 210). The criteria for exclusion were a history of hypertension or active antihypertensive medication, smokers with more than five pack year history, heavy alcohol use (defined as per the National Institute on Alcohol Abuse and Alcoholism (NIAAA) guidelines), and self-reported sleep time of less than seven hours or more than nine hours/day.

Exclusion criteria

Hypertension

We used the guidelines laid down by the European Society of Cardiology (ESC) for the assessment of BP. Values ≥130 mmHg (systolic) or ≥85 mmHg (diastolic) were considered elevated. Individuals with a history of hypertension or on active anti-hypertensive medication were excluded from the study (n = 4).

Smoking

Individuals were asked about their smoking status by means of a comprehensive questionnaire. For excluding smokers, more than five pack years was used as the cutoff [7]. Smokers constituted 9.52% (n = 20) of the study population. None of the individuals were excluded from this study based on their pack year history.

Alcohol

The NIAAA defines heavy alcoholics as men consuming more than four drinks on any day or more than 14 drinks per week and women consuming more than three drinks on any day or more than seven drinks per week [8]. A total of 19.7% (n = 40) individuals out of the total study population (n = 210) reported alcohol consumption. None of the individuals reported heavy alcohol use and hence were not excluded from the study.

Sleep Duration

Individuals with self-reported sleep of less than seven hours or more than nine hours per day were excluded from the study.

Covariates

Level of physical activity and BMI were potential confounders and hence included in the present analysis.

Data collection and measurements

Blood Pressure

BP was recorded from the left arm in a sitting position using the auscultatory method. Two BP readings were taken in a designated chamber using a standardized sphygmomanometer. Individuals were asked to avoid any caffeinated or tobacco products on the day of BP measurement. They were sat for 15 minutes comfortably in a room with a temperature of 27℃ before the first reading was taken. There was a gap of two minutes before the second reading was recorded. Systolic BP was recorded at the first Korotkoff sound and diastolic BP was the point of the last audible Korotkoff sound. The higher of the two readings was used to classify elevated BP.

Assessment of Screen Time

Screen time refers to the cumulative average daily time spent on smartphones, tablets, laptops, televisions, and computers.

The data on screen time was followed over a period of three weeks and averaged out for one day. Both iOS and Android device users were asked to share their screen time data, which is in-built in the device operating system. Television or laptop time was self-reported for those who could not obtain the data from device management.

Data were analysed by taking cutoffs for screen time as 240 minutes (four hours), 360 minutes (six hours), 390 minutes (six hours and 30 minutes), 420 minutes (seven hours) and 480 minutes (eight hours).

Physical Activity

Energy expenditure was expressed by multiples of the metabolic equivalent of tasks (METs), as shown in Table 1.

**Table 1:** Classification of physical activity based on METs One metabolic equivalent of task (MET) is defined as the amount of oxygen consumed while sitting at rest and is equal to 3.5 ml O2 per kg body weight x minutes.

Metabolic equivalent of tasks (METs)	Absolute intensity
1 to 1.5	Sedentary
1.6 to <3.0	Light intensity activity
3.0 to <6.0	Moderate-intensity activity
6.0 or higher	Vigorous intensity activity

Data on the individual’s level of physical activity were self-reported by means of a comprehensive questionnaire. In our study, individuals with sedentary to light-intensity activity (METs < 3) were compared to individuals with moderate to high-intensity activity (METs > 3).

Assessment of Socio-Economic Scale

The modified Kuppuswamy scale was used to stratify individuals according to their socioeconomic status (SES).

Study methodology and statistical analysis

Data were entered into Microsoft Excel (Microsoft® Corp., Redmond, WA) and analysed using the latest SPSS version 29 software (IBM Corp., Armonk, NY). To check the normality of the data, the Kolmogorov-Smirnov/Shapiro-Wilk test was applied. Linear correlation between the quantitative variables was analysed using Spearman rank correlation for non-normally distributed data. Multiple linear regression analysis was used to assess the association of total screen time with BP and BMI. The role of mediators in the observed association was also examined. The null hypothesis was that no correlation existed between screen time and BP. The significance level was set at 5%. A p-value < 0.05 would lead to the rejection of the null hypothesis.

## Results

Prospective analysis

Students (n = 210) across four batches were stratified according to their year of admission. Following the ESC guidelines, we observed that 35.8% of students had BPs either in the high normal range or were hypertensive. Any value ≥ 130 mmHg (systolic) or ≥ 85 mmHg (diastolic) was considered elevated.

On the evaluation of our study population, 135 students (64.2%) had normal BP, 30 (14.2%) had elevated diastolic BP, 32 (15.2%) had elevated systolic BP, and 13 (6.1%) had elevated systolic as well as diastolic BP. The baseline characteristics of our study population are mentioned in Table 2.

**Table 2:** Baseline characteristics ST: screen time; SD: standard deviation; SE: standard error; BP: blood pressure.

Sex	N	Percentage (%)
Male	158	75.23
Female	52	24.76
Total	210	
Student batch (year-wise)	N	Percentage (%)
2018	44	20.95
2019	55	26.19
2020	55	26.19
2021	56	26.66
Total	210	
Characteristics	N ± SD	SE
Mean age (years)	21.4 ± 1.6	0.115
Mean BMI (kg/m^2^)	23.15 ± 3.33	0.229
Mean ST (minutes)	402.24 ± 157.21	10.85
Mean BP	N ± SD (mmHg)	SE
Systolic BP 1	122.24 ± 9.79	0.675
Systolic BP 2	121.61 ± 9.77	0.674
Diastolic BP 1	76.59 ± 10.08	0.695
Diastolic BP 2	76.93 ± 9.81	0.677

Screen time and blood pressure

Spearman’s correlation analysis of screen time and BP showed no significant findings with a p-value > 0.05. The screen time data were analysed with both systolic and diastolic readings. Results were insignificant when screen time was correlated with systolic reading 1 (p = 0.818), systolic reading 2 (p = 0.771), diastolic reading 1 (p = 0.385), and diastolic reading 2 (p = 0.505).

For further analysis, individuals were classified into screen time ≥240 minutes (four hours), ≥360 minutes (six hours), ≥390 minutes (six hours and 30 minutes), ≥420 minutes (seven hours), and ≥480 minutes (eight hours). The odds of having elevated BP (≥130 mmHg systolic or ≥ 85 mmHg diastolic) were analysed at different screen times. The findings are mentioned below (Table 3).

**Table 3:** Odds ratio of elevated BP for different screen times * Correlation is statistically significant (p < 0.05). ST: screen time; CI: confidence interval; BP: blood pressure.

ST ≥ minutes	Odds ratio	95% CI	Z statistic	P-value
≥240	1.1786	0.48-2.86	0.362	0.717
≥360	1.7082	0.96-3.03	1.827	0.0677
≥390	1.8657	1.05-3.30	2.141	0.0323*
≥420	1.8615	1.04-3.30	2.123	0.0338*
≥480	1.8728	1.02-3.43	2.030	0.042*

Our data showed that individuals having screen time ≥ 390 minutes had higher odds (OR: 1.10; 95% CI: 1.05-3.30) of elevated BP compared to those having screen time <390 minutes. This value was also consistent with values of screen time >420 minutes (OR: 1.86; 95% CI: 1.04-3.30) and screen time >480 minutes (OR: 1.87; 95% CI: 1.02-3.43). No such findings were observed for screen time values >360 minutes (OR: 1.70; 95% CI: 0.96-3.03) and >240 minutes (OR: 1.17; 95% CI: 0.48-2.86).

On excluding participants with high BMI (>24.9 kg/m^2^) using the WHO criteria, the analysis showed higher odds of elevated BP with screen time >360 minutes (OR: 2.10; 95% CI: 1.02-4.30), >390 minutes (OR: 3.21; 95% CI: 1.58-6.49), >420 minutes (OR: 3.20; 95% CI: 1.58-6.47), and >480 minutes (OR: 2.86; 95% CI: 1.37-5.97), as shown in Table 4.

**Table 4:** Odds ratio of elevated blood pressure for different ST with participants having BMI > 24.9 kg/m2 (WHO) excluded * Correlation is statistically significant (p < 0.05). ST: screen time; CI: confidence interval.

ST ≥ minutes	Odds ratio	95% CI	Z statistic	P-value
≥240	1.3026	0.39-4.31	0.433	0.6654
≥360	2.1018	1.02-4.30	2.030	0.0423*
≥390	3.2117	1.58-6.49	3.244	0.0012*
≥420	3.2076	1.58-6.47	3.252	0.0011*
≥480	2.8636	1.37-5.97	2.805	0.005*

On excluding participants with high BMI (>22.9 kg/m2) using the Asia-Pacific criteria, the analysis showed higher odds of elevated BP in participants with screen time >360 minutes (OR: 2.86; 95% CI: 1.06-7.67), >390 minutes (OR: 3.92; 95% CI: 1.49-10.31), and >420 minutes (OR: 3.54; 95% CI: 1.38-9.10), as shown in Table 5. However, the odds ratio was insignificant at screen time >480 minutes (eight hours), which could be due to less number of participants in that group.

**Table 5:** Odds ratio of elevated BP for different screen times with participants having BMI > 22.9 kg/m2 (Asia-Pacific) excluded * Correlation is statistically significant (p < 0.05). ST: screen time; CI: confidence interval; BP: blood pressure.

ST ≥ minutes	Odds ratio	95% CI	Z statistic	P-value
≥240	1.5923	0.32-7.92	0.568	0.5698
≥360	2.8653	1.06-7.67	2.094	0.0362*
≥390	3.9231	1.49-10.31	2.772	0.0056*
≥420	3.5455	1.38-9.10	2.631	0.0085*
≥480	2.6222	0.98-6.99	1.927	0.0540

Based on these findings, high screen time was defined as >390 minutes (six hours and 30 minutes), as the odds ratio was consistent at this value across all analyses with or without correction for high BMI. It was also found that the prevalence of elevated BP (≥130 mmHg systolic or ≥85 mmHg diastolic) was higher in students having screen time >390 minutes (43.61%) compared to students having screen time <390 minutes (29.31%).

Physical activity and screen time

To identify the relationship between screen time and physical activity in our study population, the odds ratio was calculated between screen time with exposure as ≥390 minutes or <390 minutes and outcome was identified as either sedentary-light physical activity (METs < 3) or moderate-high intensity physical activity (METs ≥ 3). The results were insignificant with a p-value > 0.05.

The findings are similar to the study conducted by O'Brien et al., which showed no significant interaction when examining daily physical activity and overall screen time in the prediction of early adolescents’ BMI [9].

Physical activity and blood pressure

It has been shown that regular exercise can decrease left ventricular mass in hypertensive adults and even lower the average systolic and diastolic BP [10].

Hence, it was important to identify the level of physical activity in our study population and compare it with BP readings as this could be a potential confounder. The outcome was identified as elevated or normal BP and exposure was identified as METs <3 or ≥3. Results were insignificant (p > 0.05).

BMI and elevated blood pressure

It was found that a strong gradient exists between increasing BP with higher levels of BMI (p < 0.001). The fact that this gradient is present even in the fully adjusted analyses suggests that BMI may cause a direct effect on BP, independent of other clinical risk factors. The results of our study are consistent with the Longevity Check-Up 7+ trial and come as a parallel finding [11].

Screen time and number of devices

The number of devices used was identified as one, two, three, or more than three devices. Spearman’s rho correlation analyses between the number of electronic devices and total screen time showed a positive correlation with a p-value of 0.035 (Table 6). This gives us the impression that higher screen times were associated with a greater number of devices used by the individuals.

**Table 6:** Correlation between screen time and number of devices * Correlation is significant at the 0.05 level (two-tailed).

			Screen time	Devices
Spearman's Rho	Screen time	Correlation coefficient	1	0.146*
		Sig. (2-tailed)	-	0.035
		N	210	210
	Devices	Correlation coefficient	0.146*	1
		Sig. (2-tailed)	0.035	-
		N	210	210

On comparing the demographic data of our study population, high SES individuals used more devices on average, compared to lower SES (with two devices being the most popular choice in higher SES and one device being the most popular choice in lower SES).

## Discussion

We found that individuals with screen time >390 minutes (six hours and 30 minutes) had higher odds of elevated BP (p < 0.05) with or without correction for high BMI. It was also evident that having a greater number of devices made it more likely for people to spend more time using them. The prevalence of elevated BP (≥130 mmHg systolic or ≥85 mmHg diastolic) in our sample population is 35.8%, which is similar to the findings of Anchala et al. [5]. This is an alarming statistic and raises a reasonable concern for the general health and well-being of young adults.

Another significant finding in our study is the strong correlation between BP and BMI (p < 0.001), suggesting that a gradient exists between increasing BP with higher levels of BMI.

On exploring existing data, Nang et al. showed in their study that longer TV screen time was significantly associated with higher systolic BP after adjustment for potential socio-demographic and lifestyle confounders [12]. Another study by Martinez-Gomez et al. showed that excessive TV viewing (> three hours/day) was related to an unfavourable cardiovascular disease risk factor profile in adolescence [13]. Mark et al. in a study on adolescents found that screen time was associated with an increased likelihood of metabolic syndrome (MetS) in a dose-dependent manner independent of physical activity [14]. However, these studies relied on self-reported screen time data rather than close follow-up.

In exploring the potential risk factors for hypertension, Parthaje et al. suggested that increasing age, male gender, lack of physical activity, obesity, tobacco and alcohol use, and family history of chronic diseases were associated with prehypertension and hypertension [15].

The American Academy of Sleep Medicine and Sleep Research Society suggest most adults receive sleep between seven and nine hours per night. A prospective study of patients with prehypertension who slept less than seven hours per night showed decreased systolic and diastolic BP by 14 mmHg and 8 mmHg, respectively, if sleep duration was increased by approximately 35 minutes/night over six weeks [16]. Moreover, findings from the Sleep Heart Health Study showed that individuals sleeping more than nine hours per night had a 30% greater risk of hypertension relative to those sleeping between seven and eight hours [17]. For the purpose of our study, we excluded participants who slept for less than seven hours or more than nine hours/day.

Undoubtedly, several factors affect the BP of an individual, out of which having a higher screen time, associated with increased stress levels, poor sleeping habits, and decreased level of physical activity, may be a significant one. High screen time at night has been proven to hamper sleep quality and impair cognitive function [18-20].

Moreover, the diastolic BP may be more sensitive to changes in screen time, possibly due to vascular changes because of higher stress levels associated with excessive screen usage, as indicated by the higher prevalence of elevated diastolic BP (55.8%) compared to elevated systolic BP (48.8%) in individuals with high screen time. However, much cannot be commented on solely based on a cross-sectional analysis, and this serves as an area of future research and exploration.

Strengths of our study

We closely followed up participants’ screen time for a period of three weeks rather than relying on self-reported screen time.

Limitations of our study

Although physical activity and BMI were considered in the present analysis, eating habits were not.

## Conclusions

Our study found that individuals with screen time >390 minutes (six hours and 30 minutes) had higher odds of elevated BP (p < 0.05) with or without correction for high BMI. It was also evident that having more devices made it more likely for people to spend more time using them. Another significant finding in our study is the strong correlation between BP and BMI (p < 0.001), suggesting that a gradient exists between increasing BP with higher levels of BMI.
